# Alpha-fetoprotein-producing rectal cancer successfully responded to preoperative chemoradiotherapy: case report

**DOI:** 10.1186/s40792-018-0520-6

**Published:** 2018-09-06

**Authors:** Yuki Nakamura, Kenji Matsuda, Shozo Yokoyama, Koichi Tamura, Yasuyuki Mitani, Hiromitsu Iwamoto, Yuki Mizumoto, Daisuke Murakami, Masakazu Fujimoto, Hiroki Yamaue

**Affiliations:** 1 Second Department of Surgery, School of Medicine, Wakayama Medical University, 811-1, Kimiidera, Wakayama, 641-8510 Japan; 2 Department of Human Pathology, School of Medicine, Wakayama Medical University, Wakayama, Japan

**Keywords:** Alpha-fetoprotein (AFP), Rectal cancer, Chemoradiotherapy

## Abstract

**Background:**

Alpha-fetoprotein (AFP) is produced by some tumors, such as hepatocellular carcinoma and yolk sac tumors, leading to an increase in serum AFP level. However, AFP in colorectal cancer is extremely rare. Treatment for AFP-producing cancer is often performed according to conventional methods, but oncological outcomes of both surgery and chemotherapy are poor. We report a case of a patient with AFP-producing rectal cancer which successfully responded to preoperative chemoradiotherapy.

**Case presentation:**

Rectal tumor was diagnosed in a 68-year-old man referred to our hospital. Colonoscopy showed a type 2 tumor in the lower rectum, and biopsy revealed an adenocarcinoma with enteroblastic differentiation. Serum tumor marker levels were 8.8 ng/ml in carcinoembryonic antigen (CEA) and 28.3 ng/ml in AFP. Clinical diagnosis was stage IIIB (T3N1M0), and preoperative chemoradiotherapy was performed to prevent local recurrence. Effective tumor reduction was observed, and serum tumor marker levels decreased to normal range. Low anterior resection with temporary diverting ileostomy was performed, and histology revealed residual adenocarcinoma. Pathological diagnosis was stage I (T2N0M0). The tumor was found to be an AFP-producing adenocarcinoma on further immunohistopathological examination. The postoperative course was uneventful, and the patient received adjuvant chemotherapy for 3 months.

**Conclusions:**

The outcomes of preoperative chemoradiotherapy against AFP-producing rectal cancer are reported here for the first time. Based on our experience with this patient, it appears preoperative chemoradiotherapy for patients with AFP-producing advanced rectal cancer is feasible.

## Background

Alpha-fetoprotein (AFP) is an oncofetal glycoprotein produced from fetal liver cells, yolk sac cells, and in small quantity from fetal gastrointestinal epithelial cells [1]. In some tumors, such as hepatocellular carcinoma and yolk sac tumors, AFP is produced, which leads to an increase in serum AFP level. It is therefore commonly used as a tumor marker for diagnosis and monitoring of treatment [2, 3]. AFP production from tumors of other organs such as the stomach, the bile duct, and the pancreas has also been reported [4, 5].

However, it is extremely rare that AFP is produced in colorectal cancer. Treatment is often performed conventionally, but oncological outcomes of both surgery and chemotherapy are poor. It is generally associated with a poor prognosis because of the high frequency of liver metastasis [6].

Here, we report a case of AFP-producing colorectal cancer which successfully responded to chemoradiotherapy. The effectiveness of preoperative chemoradiotherapy against the AFP-producing colorectal cancer is reported here for the first time.

## Case presentation

**Fig. 1:**
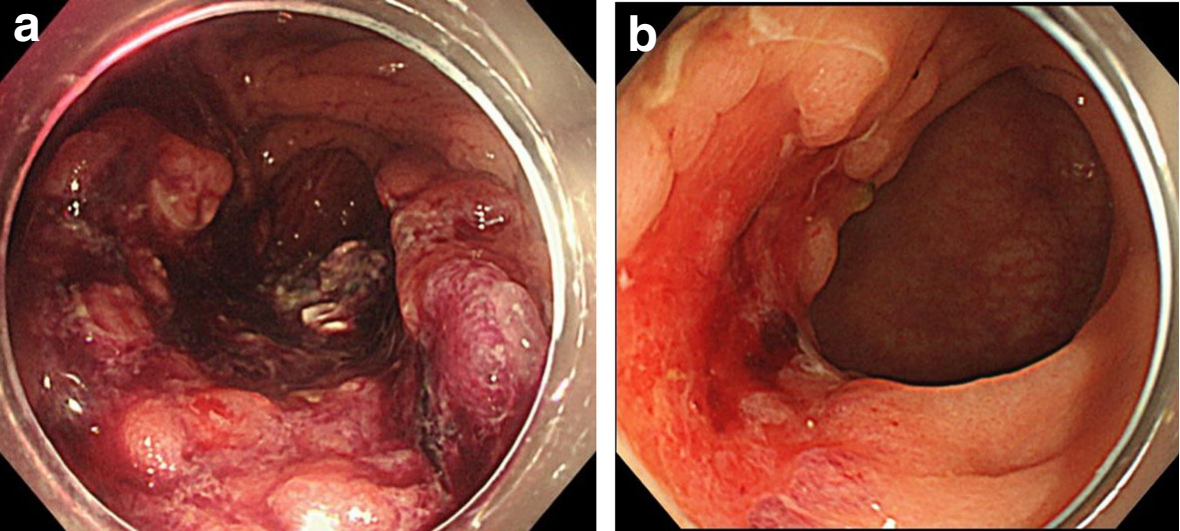
Colonoscopy findings. Colonoscopy revealed a type 2 tumor in the rectum (**a**). Five weeks after chemoradiotherapy, the tumor was significantly reduced in size (**b**)

**Fig. 2:**
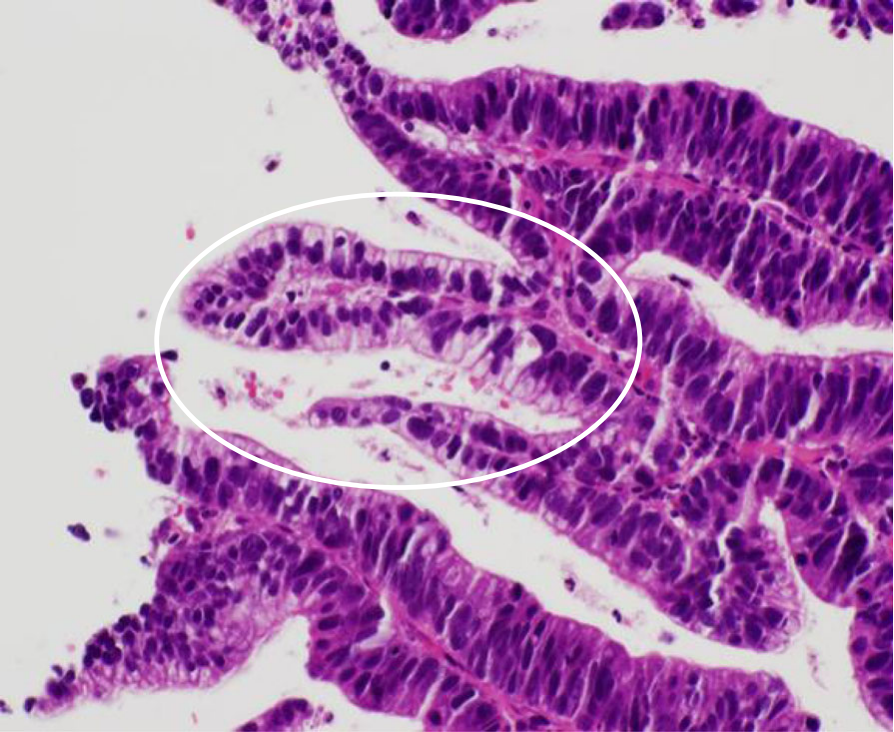
A biopsy finding. Biopsy showed an adenocarcinoma with enteroblastic differentiation component (circle). (H.E. × 200)

**Fig. 3:**
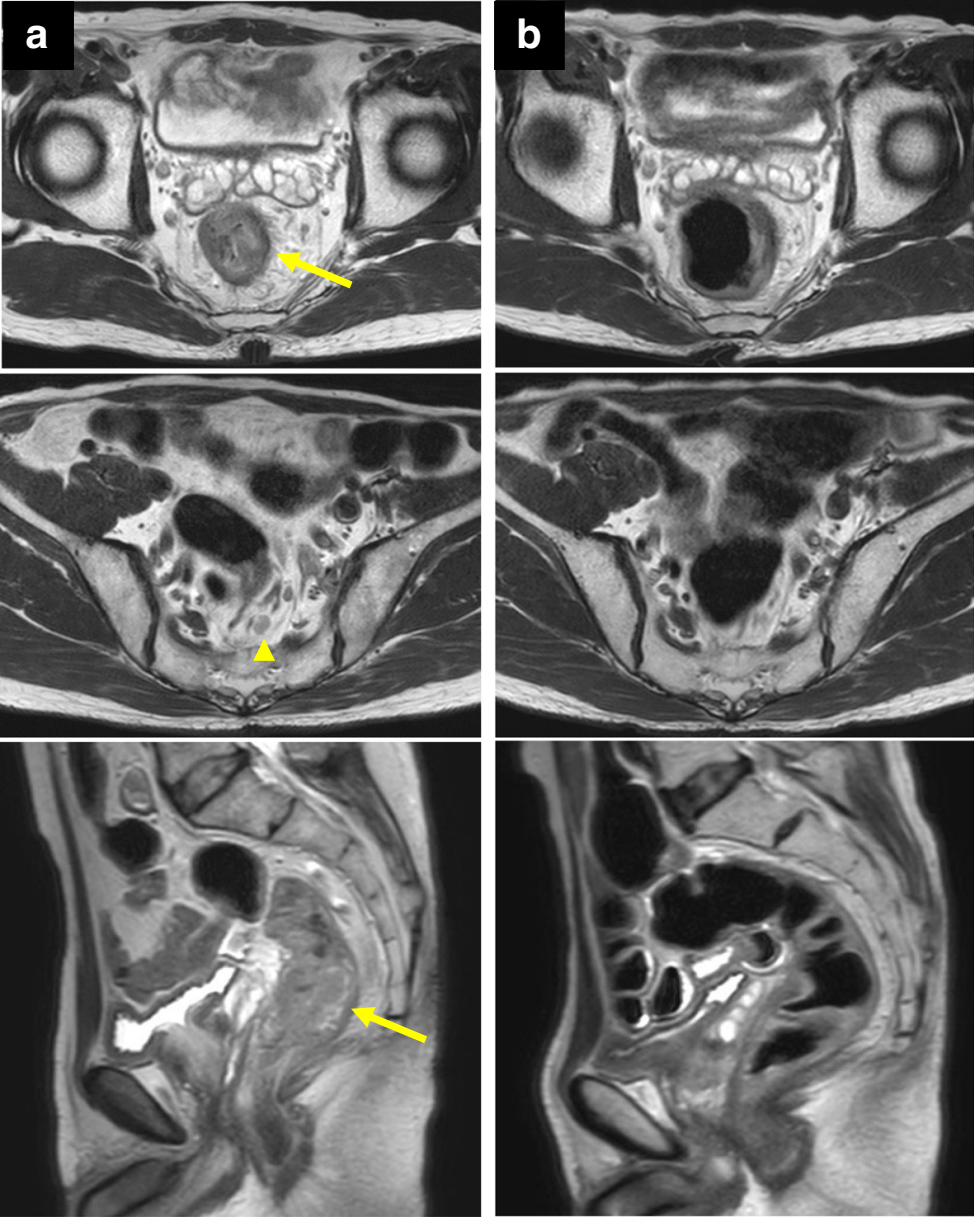
MRI findings. Pelvic MRI showed a large tumor in the rectum (arrow) and a pararectal lymph node swelling (arrow head) (**a**). Five weeks after chemoradiotherapy, they were remarkably shrunk (**b**)

**Fig. 4:**
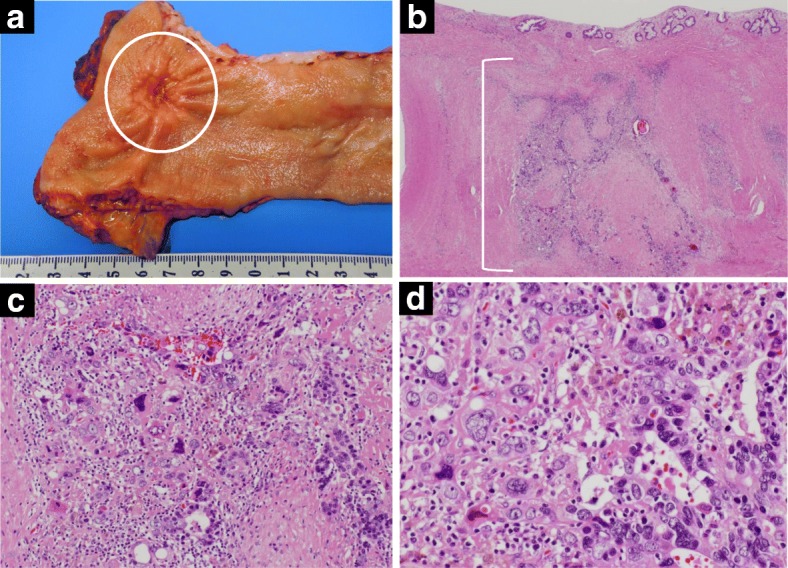
Macroscopic and microscopic findings. Macroscopic findings of the resected specimen revealed an ulcerative lesion measuring 2.0 cm × 2.0 cm (**a**). Microscopic findings showed residual poorly differentiated adenocarcinoma with degeneration caused by radiation therapy. (**b** H.E. × 20, **c** H.E. × 100, **d** H.E. × 200)

**Fig. 5:**
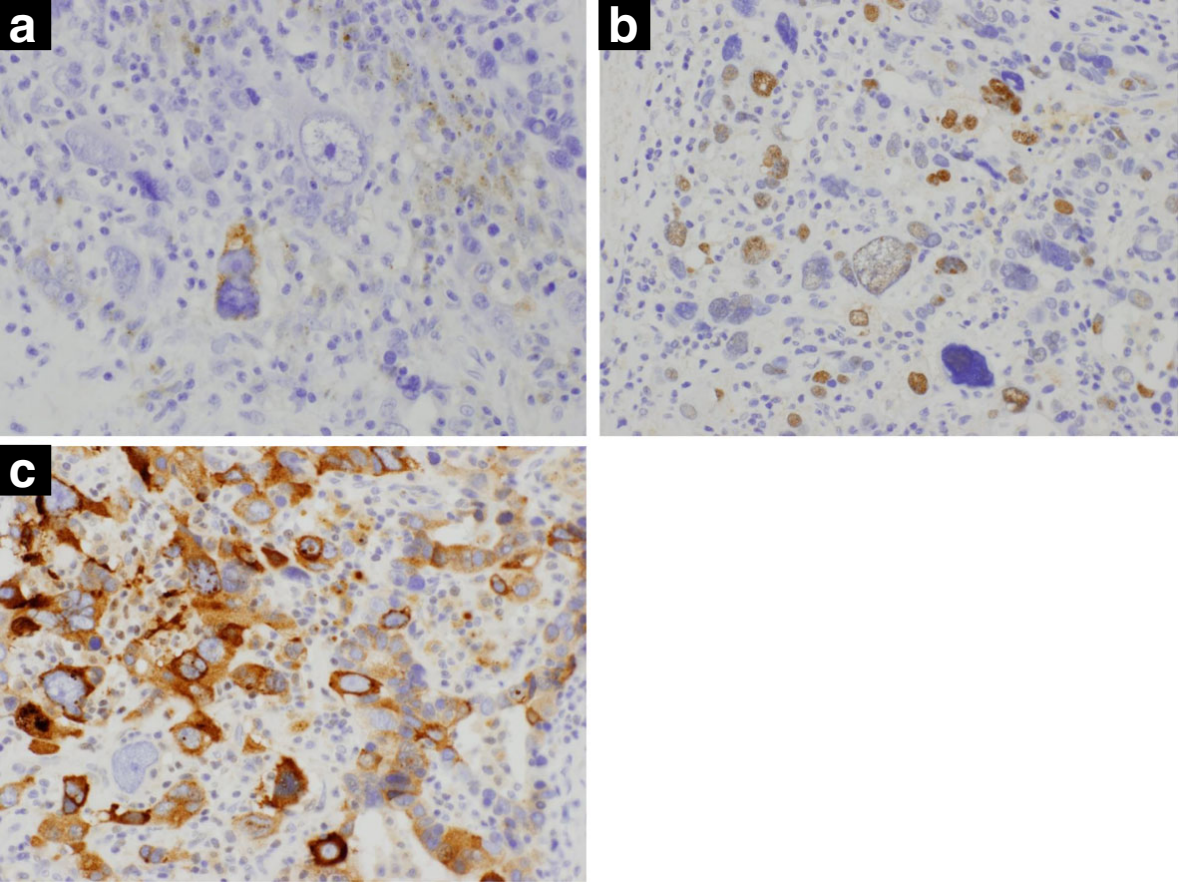
Immunohistochemical findings. Immunohistochemical studies yielded positive results for AFP, SALL4, and GPC3. (**a** AFP × 200, **b** SALL4 × 200, **c** GPC3 × 200)

A 68-year-old man was referred to our hospital with diagnosis of rectal tumor. Medical history notably included diabetes mellitus, but family and social history were unremarkable. Colonoscopy identified a two-thirds circumferential type 2 tumor in the rectum, about 5 cm from the anal verge (Fig. 1a). Biopsy of the tumor revealed well differentiated tubular adenocarcinoma and papillary adenocarcinoma with enteroblastic differentiation which was characterized by clear cytoplasm and regarded as one of the histological features in AFP-producing cancer (Fig. 2). Laboratory evaluation showed fasting blood glucose and HbA1c levels were elevated at 152 mg/dl (normal range 73–109 mg/dl) and 13.7% (normal range 4.9–6.0%), respectively. Serum tumor marker levels were increased to 8.8 ng/ml in CEA (normal range ≤ 5.0 ng/ml) and 28.3 ng/ml in AFP (normal range ≤ 7.0 ng/ml). Two-thirds circumferential thickening of the wall over 4 cm in the lower rectum, and a pararectal lymph node swelling about 8 mm in diameter was revealed by enhanced abdominal computed tomography (CT) and pelvic magnetic resonance imaging (MRI) (Fig. 3a). The tumor was classified as stage IIIB (T3N1M0). To improve the local control rate and the survival rate, preoperative radiation therapy (total dose of 45 Gy/25 fractions) with capecitabine (1,650 mg/m^2^/day) was performed. Effective tumor reduction was observed on colonoscopy, CT, and MRI after 5 weeks of the above treatment. A swelled pararectal lymph node also showed a significant decrease of its size from 8 to 3 mm in diameter (Fig. 1b, 3b). In addition, serum tumor marker levels decreased to normal range: CEA, 2.0 ng/ml; AFP, 3.7 ng/ml. At 7 weeks, low anterior resection with temporary diverting ileostomy was performed. Histopathologically, residual poorly differentiated, non-solid type adenocarcinoma was present, although most of the tumor comprised fibrous scar tissue. There was no lymph node metastasis, and pathological diagnosis was stage I (T2N0M0). Histological evaluation of the treatment with chemoradiotherapy was assessed to be grade 2 according to the Japanese Classification of Colorectal Carcinoma [7]. Immunohistochemical studies yielded positive results for AFP, Sal-like protein 4 (SALL4), and glypican3 (GPC3) (Figs. 4, 5). The postoperative course was uneventful. Four weeks after the operation, serum tumor marker levels had decreased to 1.2 ng/ml in CEA and 1.6 ng/ml in AFP. The patient received adjuvant chemotherapy with capecitabine and oxaliplatin (CAPOX) for 3 months in consideration of high　recurrence rate in AFP-producing cancer. After completing this regimen, we checked no signs of recurrence. To date, he has not developed any recurrence for 6 months after the operation.

### Discussion

AFP is a serum glycoprotein frequently detected in patients with hepatocellular carcinoma and yolk sac tumors [1–3]. Its production has also been reported in malignant tumors of various organs, such as the bile duct, the pancreas, and particularly the stomach [4, 5]. AFP-producing colorectal cancer, however, is extremely rare. AFP-producing gastric cancers are associated with aggressive clinical behavior and poorer prognosis compared to AFP-negative gastric cancer because of a significantly higher incidence of vascular invasion, lymph node metastasis, and liver metastasis [6, 8, 9]. As for AFP-producing colorectal cancers, a similar tendency has been observed in previous reports and their reviews of up to 12 patients [10–13]. Patients with AFP-producing colorectal cancer underwent several treatments, including surgery and chemotherapy according to conventional colorectal cancer treatment. There are no confirmed treatment strategies, however, and about half of the patients died within a year of therapeutic intervention. The present case was of rectal cancer classified as stage IIIB (T3N1M0), so we elected to perform the preoperative chemoradiotherapy that is recommended for cases clinically diagnosed as deeper than T3 or node-positive rectal cancer. The aim was to improve the local control rate and the survival rate [14–17]. As of chemotherapy regimen for preoperative chemoradiotherapy, we chose capecitabine alone according to the papers describing that preoperative radiation therapy with capecitabine was as effective as with intravenous infusional fluorouracil, but the addition of oxaliplatin did not improve surgical and oncological outcomes [18, 19].

Although there are no reports of AFP-producing rectal cancer treated with radiation therapy, several reports in hepatocellular carcinoma and a few reports in yolk sac tumor have shown its effectiveness [20–22]. In our case, effective tumor reduction was observed and serum tumor marker levels decreased to normal range by this treatment. After 7 weeks of the above treatment, the operation was performed. Although pathological findings showed R0 resection, immunohistochemical studies revealed AFP production. The tumor was therefore diagnosed as AFP-producing adenocarcinoma. Immunohistochemical studies also yielded positive results for SALL4 and GPC3; known novel oncofetal proteins expressed in germ cell tumors. SALL4 and GPC3 are also highly expressed in AFP-producing gastric cancers [23], but there are no reports on their expression in AFP-producing colorectal cancers. In this case, as with AFP-producing gastric cancers, fetal differentiation may be induced, and immunohistochemical studies showed positive results for these oncofetal proteins. SALL4 expression in colorectal cancer is reportedly associated with lymph node metastasis and poor prognosis [24]. Although our case was pathologic stage I which was not normally needed for adjuvant chemotherapy in AFP-negative colorectal cancer, he was deemed to have a high probability of recurrence, and hence, adjuvant chemotherapy was performed. As effective therapies against AFP-producing colorectal cancer have not been established, regimen is performed according to various guidelines and personal experiences. Our patient received CAPOX treatment for 3 months according to the latest guidelines, and the report showing 3 months of therapy with CAPOX was as effective as 6 months in patients with stage III colon cancer even among those with high-risk factors [14, 25].

## Conclusion

In our patient, AFP-producing rectal cancer responded to preoperative chemoradiotherapy, and R0 resection was achieved. The effectiveness of preoperative chemoradiotherapy for AFP-producing rectal cancer is reported here for the first time. It may be feasible to perform preoperative chemoradiotherapy for patients with AFP-producing advanced rectal cancer.

## Abbreviations

AFP: Alpha-fetoprotein
CT: Computed tomography
GPC3: Glypican3
MRI: Magnetic resonance imaging
SALL4: Sal-like protein 4
